# The epidemiological trends and projected future of primary sclerosing cholangitis by 2040: An updated meta-analysis and modeling study

**DOI:** 10.1371/journal.pone.0322479

**Published:** 2025-05-05

**Authors:** Meysam Olfatifar, Mohsen Rajabnia, Amir Sadeghi, Amirhassan Rabbani, Shabnam Shahrokh, Mohammad Amin Habibi, MehdiSure Pezeshgi Modarres, Mohammad Reza Zali, Hamidreza Houri

**Affiliations:** 1 Gastroenterology and Hepatology Diseases Research Center, Qom University of Medical Sciences, Qom, Iran; 2 Non-Communicable Diseases Research Center, Alborz University of Medical Sciences, Karaj, Iran; 3 Gastroenterology and Liver Diseases Research Center, Research Institute for Gastroenterology and Liver Diseases, Shahid Beheshti University of Medical Sciences, Tehran, Iran; 4 Department of Transplant & Hepatobiliary Surgery, Shahid Beheshti University of Medical Sciences,Tehran, Iran; 5 Clinical Research Development Center, Qom University of Medical Sciences, Qom, Iran; 6 Foodborne and Waterborne Diseases Research Center, Research Institute for Gastroenterology and Liver Diseases, Shahid Beheshti University of Medical Sciences, Tehran, Iran; King Saud University Medical City, SAUDI ARABIA

## Abstract

**Background and Aims:**

Primary sclerosing cholangitis (PSC) exhibits varying incidence and prevalence rates across different regions; however, comprehensive global studies examining its geographic distribution and future trends are scarce. This study presents an updated meta-analysis through 2024 and projects the global and regional prevalence of PSC from 2024 to 2040 using an illness-death multi-state model.

**Methods:**

We conducted a thorough systematic search across multiple databases to identify all primary studies published until 2024 that reported on the incidence, prevalence, and mortality rates of PSC in various regions. Using the gathered data, we developed an illness-death model to forecast the future prevalence of PSC, covering the years 2024–2040.

**Results:**

Our meta-analysis revealed that the global pooled incidence and prevalence rates of PSC are 0.65 and 7.52 per 100,000 persons, respectively. Projections indicate that the global prevalence of PSC will rise to 22.98 cases per 100,000 (95% CI: 21.0–24.95), corresponding to an overall increase of 28.3%. Specifically, North America is forecasted to experience a 5.45% increase in PSC cases, reaching 24.76 cases per 100,000 (95% CI: 19.63–29.88), while Western Europe is anticipated to see a more pronounced rise of 28.79%, resulting in a prevalence of 21.48 cases per 100,000 (95% CI: 18.3–24.65) by 2040.

**Conclusions:**

Our findings indicate a substantial rise in the number of individuals affected by PSC in recent years and estimate a significant future burden of the disease.

## Introduction

Primary sclerosing cholangitis (PSC) is a rare, idiopathic, and progressive cholestatic liver disorder that may lead to hepatic fibrosis and cirrhosis [1]. The biliary affection in PSC is chronic and represented by mild inflammation and progressive concentric fibrosis (‘onion skin’ fibrosis) around the intrahepatic and/or extrahepatic bile ducts [2]. Clinical manifestations reflect the underlying sequence of bile duct injury and fibrosis over the course of one to two decades leading to biliary strictures, cholestasis, and cirrhosis with progressive liver dysfunction [2]. It is estimated that roughly 70% of patients with PSC have underlying inflammatory bowel disease (IBD) [3]. It has been also reported that PSC is typified by a strong association with ulcerative colitis (UC), however, few patients also exhibit clinical features of Crohn’s disease (CD) [4]. Accordingly, procedures for early, potentially pre-clinical diagnosis of PSC are usually restricted to UC patients in whom abnormal liver biochemistry suggests the presence of liver dysfunction [1].

Although the etiology and pathogenesis of PSC still remain a scientific and clinical challenge, the interplay of multiple genetic variants and environmental factors has been described to be implicated in PSC development [5]. Based on the associations of PSC with multiple autoantibodies (autoAbs), human leukocyte antigen complex (HLA) haplotypes, and the presence of IBD in the majority of PSC patients, immunopathogenic mechanisms have been suggested to contribute to the onset of this condition [6]. To date, there are no approved pharmacological therapies for PSC, and liver transplantation offers a curative and life-prolonging treatment for patients with decompensated cirrhosis and hilar cholangiocarcinoma caused by PSC, however, recurrent PSC is estimated to occur in approximately 20% of the patients [7].

The prevalence and incidence of PSC vary in various regions globally, with the highest rates generally observed in Northern Europe [8]. A meta-analysis conducted in 2021 found that the prevalence of PSC varied between 0 and 31.7 cases per 100,000 individuals, which appears to remain constant over time. Accordingly, it has been reported that the highest prevalence was 31.7 per 100,000 persons in Finland and the lowest prevalence was 0 per 100,000 persons in Alaska [9]. The mean incidence rate of PSC has also been estimated to be 0.6 per 100,000 person-year, ranging from 0.04 to 1.58 per 100,000, and may be increasing in Northern Europe and North America [9]. However, the prevalence and incidence of PSC seem to be about ten‑fold lower in Asian regions [10]. Moreover, the incidence rate of PSC varies with gender and age occurring twice as frequently in males (1.25 vs 0.54 per 100,000 person-years) and is most often diagnosed between the ages of 30 and 40 years [11–13].

Importantly, the majority of PSC epidemiological research has been conducted based on limited case series or small population-based subsets of European and North American regions, and hence, there is a paucity of data regarding inter-regional associations of PSC. Furthermore, the increasing number of IBD cases could have a substantial influence on the global burdens of PSC. Therefore, it is beneficial to forecast the epidemiological characteristics of this condition by considering its geographic distribution. Such predictions can aid in the development of public health initiatives aimed at alleviating the global burdens associated with PSC. This study offers new insights and improvements to previous research on the epidemiology of PSC with some innovations until September 2024. In addition, we present the first global, regional, and national PSC-related mortality rates that have not been reported in previous studies. We projected the further prevalence of PSC around the world by 2040 using an illness-death multi-state model.

## Methods

### Ethics approval and consent to participate

Not applicable.

#### Literature search strategy and study selection.

The systematic review and meta-analysis, conducted according the Preferred Reporting Items for Systematic Review and Meta‐Analyses (PRISMA) statement [14]. Accordingly, we systematically searched the medical literature using searching Medline (via PubMed), Embase, Cochrane Library for Systematic Reviews, and Web of Science, to retrieve available data on PSC epidemiology. The search strategy we implemented encompassed English-language publications from 1970 to September 2024. We employed the Medical Subject Heading (MeSH) terms ‘primary sclerosing cholangitis’ OR ‘sclerosing cholangitides’ OR ‘sclerosing cholangitis’ were combined using the set operator AND with the terms ‘epidemiology’ OR ‘prevalence’ OR ‘incidence’ OR ‘mortality’ OR ‘morbidity’ OR ‘surveillance’ to ensure comprehensive coverage of relevant literature pertaining to the study. For selecting articles, two reviewers were consulted simultaneously, and in cases where they did not have a shared opinion, a third reviewer was consulted for assistance. To select eligible articles, the first step involved removing duplicate articles. Subsequently, we assessed the titles and abstracts of the remaining articles, and finally, we thoroughly examined the full texts of eligible articles while excluding those that did not meet the criteria. Inclusion criteria consisted of all original research studies reporting on PSC and/or prevalence incidence rates published since 1970. The conditions for exclusion were: (i) if the research did not report original data; (ii) if the presented data overlapped with other populations; (iii) if the study reported a zero incidence and/or prevalence; and (iv) if the research is conducted on a specific population, such as patients with comorbid IBD. To effectively manage missing data in our study, primarily concerning essential outcomes such as incidence, prevalence, and mortality rates for PSC, we implemented two key strategies. In cases where reports provided figures on prevalent/incident cases and derived prevalence/incidence rates but lacked information on the at-risk population or denominators, we resorted to estimating the missing denominator by recalculating using pertinent incidence/prevalence formulas. Furthermore, recognizing the significance of resolving data discrepancies promptly, we proactively engaged with corresponding authors to facilitate accurate data acquisition and analysis. These adjustments and interventions were crucial in enhancing the robustness and reliability of our study findings, underscoring our commitment to meticulous data handling and methodological rigor.

#### Publication bias and Hetrogenesitu assessment.

To identify potential publication bias, Egger’s test alongside the Luis Furuya-Kanamori (LFK) index was used. An LFK index close to ± 1 indicates no significant asymmetry, while values between ±1 and ± 2 represent minor asymmetry, and an LFK index exceeding ±2 signifies major asymmetry. The inconsistency index (I 2 statistics) was used to evaluate the magnitude of heterogeneity among included studies, considering I 2 values of 0–25% as low, 25–50% as moderate, and 50–75% as high heterogeneity

#### Model description.

In this study, we used the illness-death model (IDM) to project the future prevalence of PSC, which constitutes a holistic strategy that relies on a customized multi-state model and serves as an instrument to appraise the interplay among incidence, prevalence, and mortality. These models can be used to model the transition of patients among the various states over age and/or time for a particular process. Specifically, patients are assigned to one of three states - healthy, sick, or deceased - depending on their clinical status. The transition of patients across these states is dictated by the relative incidence and mortality rates observed in healthy versus sick/pre-symptomatic carriers of the disease. This approach enables the quantification of disease burden and aids in the evaluation of potential interventions aimed at reducing morbidity and mortality associated with the targeted condition. Importantly, we did not consider the transition back to a healthy state because it is uncommon for patients with PSC to fully recover.

#### Model assumption.

In this study, the overall incidence of PSC has been assumed to be constant over time, as it is considered acceptable for assessment of the future burden of incurable chronic diseases [15,16]. Moreover, we also assumed that the mortality rate of non-PSC healthy people and patients with PSC is constant. However, these values can be influenced by age and gender, which is inevitable in our research due to a lack of access to suitable data to populate our models. Ultimately, in cases where a country lacked specific data on mortality or incidence rates, we resorted to utilizing global estimates for mortality and regional estimates for incidence. This approach placed emphasis on regional reports as the primary source, and if a country lacked such a report, we utilized the relevant regional report that encompassed that country. It is important to note that limitations existed regarding accurate mortality rates due to a scarcity of reports specifically addressing it.

#### Model inputs.

We primarily obtained the model inputs for this study from two sources: we estimated the prevalence, incidence rate, and mortality rate of PSC worldwide using meta-analysis with a random effect model, using the meta package of R software. Moreover, we performed subgroup analysis to determine these parameters for particular regions and countries. Additionally, we obtained population size, new birth numbers, and general mortality rate (mortality rate of individuals without PSC) in 2021 from the Institute for Health Metrics and Evaluation (IHME) as the starting point for our model [17]. To populate our models, we obtained the number of PSC patients for global estimation and each region or/and country by rearranging the prevalence formula.

#### Model implementation.

We employed two deterministic and stochastic approaches to simulate IDM and predict the future prevalence of PSC at global, regional, and national scales. Initially, we populated our models using parameters sourced from existing literature. However, due to limited access to epidemiological data, we opted to utilize a system of ordinary differential equations (ODE) instead of a system of partial differential equations (PDE) in order to conduct the IDM simulations. Similarly, we used a stochastic process based on Monte Carlo to simulate IDM and estimate future PSC prevalence. In this case, based on obtaining the incidence and mortality rates, we calculate the transition probability between health states. More details on implementing these approaches are available in another similar study [18]. We used R programming software to apply both deterministic and stochastic approaches and subsequently estimate the uncertainty surrounding the results.

#### Optimization and validation.

In this study, we applied inverse problem optimization techniques to validate and improve the accuracy of our findings. These methods systematically adjust model parameters to minimize discrepancies between predicted and observed data, enabling a rigorous assessment of model performance. Specifically, we implemented the IDM using annual prevalence data for each geographical unit and optimized parameter estimates via the Limited-memory Broyden-Fletcher-Goldfarb-Shanno (L-BFGS) algorithm [19]. L-BFGS is particularly well-suited for large-scale optimization problems, as it efficiently handles high-dimensional parameter spaces while requiring minimal memory storage by retaining only a limited set of past gradients and updates. This iterative refinement process minimizes the objective function, ensuring precise parameter estimation with reduced computational overhead.

To enhance the robustness of our analysis, we restricted the evaluation to geographical units with at least three annual prevalence estimates, thereby ensuring sufficient data for reliable optimization. This criterion mitigated the risk of overfitting and strengthened the reliability of parameter estimates. By leveraging the computational efficiency of L-BFGS and implementing strict data selection criteria, we generated accurate model predictions applicable across diverse geographical regions. All the codes utilized in this study have been provided in Supporting Information File 3.

## Results

### Literature search and meta-analysis

**Fig 1:**
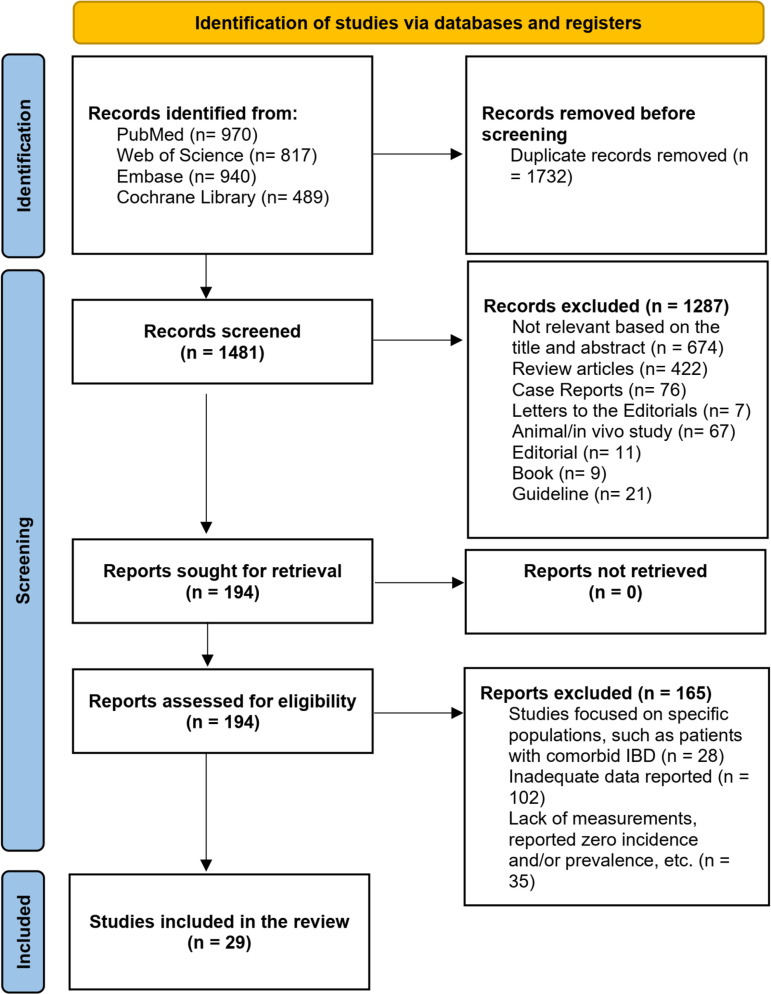
PRISMA flow diagram indicating the steps in study selection.

In our study, we used a search strategy that yielded 3216 citations. After eliminating duplicate records and review articles, we assessed 1481 records based on their titles and abstracts. Out of these, 194 records were identified as potentially relevant articles for further evaluation. Eventually, we selected 29 articles that met the eligibility criteria for data analysis and modeling (Fig 1 and S1 File). The inter-reviewer agreement for the eligibility of final records was remarkably high (0.95). The included studies spanned four geographical regions and 18 countries according to the IHME classification. Table 1 summarizes the characteristics of the included studies. The presence of publication bias was identified through Egger’s test, which showed a significant bias (t = 4.24, df = 27, *p*-value; 0.0002) with a biased estimate of 13.46 (SE = 3.17) (Supporting Information File 2 Fig S1). Furthermore, the Doi plot indicated major asymmetry, with an LFK value of 6.6 (Fig S2 in S2 File).

**Table 1:** Characteristics of the included studies on the incidence and prevalence of PSC concurrence.

Study	Publication Year	Study Period	Country (Area)	Region	Cases	Area Population	Person_Years	Death	Prevalent	Incident	Prevalence (per 100000) [95% CI]	Incidence (per 100,000)	Mortality per 100000
Escorsell *et al.* [25]	1994	1984-1988	Spain	Western Europe	43	19196429	95000000	0	43	38	0.224	0.04	–
Byron and Minuk [26]	1996	1987-1994	Canada	High-income North of America	39	650000	4239130	–	39	39	6	0.92	–
Berdal *et al*. [27]	1998	1985-1994	Norway	Western Europe	12	214286	1714286	0	12	12	5.6	0.7	–
Boberg *et al*. [28]	1998	1986-1995	Norway	Western Europe	17	200000	1297710	0	17	17	8.5	1.31	–
Ang *et al*. [29]	2002	1989-1998	Singapore	High-income Asia Pacific	10	769231	7692308	1	10	10	1.3	0.13	0.01 [0.00-0.09]
Bambha *et al*. [30]	2003	1976-2000	United States	High-income North of America	22	161765	2444444	5	22	22	13.6	0.9	0.2 [0.08-0.49]
Kingham *et al.* [31]	2004	1984-2003	United Kingdom	Western Europe	46	362205	5054945	13	46	46	12.7	0.91	0.25 [0.15-0.44]
Kaplan *et al.* [13]	2007	2000 - 2005	Canada	High-income North of America	125	1450116	5543478	–	125	51	8.62	0.92	–
Card *et al*. [32]	2008	1991-2001	United Kingdom	Western Europe	223	5792208	36341463	55	223	149	3.85	0.41	0.33 [0.11-0.19]
Lindkvist *et al*. [33]	2010	1992-2005	Sweden	Western Europe	199	1228395	16311475	29	199	199	16.2	1.22	0.17 [0.12-0.25]
Ngu *et al.* [34]	2011	1980-2008	New Zealand	Australasia	79	495726	666667		58	8	11.7	1.2	
Toy *et al*. [35]	2011	2000-2006	United States	High-income North of America	169	3573201	19756098	25	144	81	4.03	0.41	–
Boonstra *et al*. [11]	2013	2000-2007	Netherlands	Western Europe	590	9833333	118000000	73	590	590	6	0.5	0.06 [0.04-0.07]
Yanai *et al.* [36]	2015	1988-2012	Israel	Western Europe	141	3525000	106818182	15	141	141	4	0.132	–
Liu *et al*. [37]	2017	1993-2013	Australia	Australasia	208	692180	12023121	25	208	208	30.05	1.73	–
Liang *et al.* [38]	2017	1998 - 2014	United Kingdom	Western Europe	837	13676471	39062500	18	837	250	6.12	0.64	0.04 [0.02-0.07]
Gudnason *et al.* [39]	2019	1992-2012	Iceland	Western Europe	42	363243	6086957	5	42	42	11.5625	0.69	–
Tanaka *et al.* [40]	2019	2016-2016	Japan	High-income Asia Pacific	906	50333333		0	906		1.8		–
Barner-Rasmussen *et al.* [41]	2020	1990-2015	Finland	Western Europe	632	2234005	40000000	39	632	632	28.29	1.58	0.10 [0.07-0.14]
Carbone *et al.* [42]	2020	1985-2014, 2012–2014	Italy	Western Europe	502	60421169	179000000	25 and 17 lost to follow up	502	179		0.1	–
Dyson *et al.* [43]	2020	2016-2016	United Kingdom	Western Europe	472	5488372		–	472		8.6		0.235 [0.13-0.41]
Bakhshi *et al.* [44]	2020	1976-2017	United States	High-income North of America	56	233431	5045045	12	56	56	23.99	1.11	–
Deneau *et al.* [45]	2020	1986-2011 Limited to 2005–2011	United States	High-income North of America	29	1933333	14500000	1, only psc0	29	29	1.5	0.2	–
Lamba *et al.* [46]	2021	2008-2016	New Zealand	Australasia	47	356872	5108696	–	47	47	13.17	0.92	0.02 [0.01-0.03]
Tan *et al.* [47]	2022	2000-2020	Australia	Australasia	413	7000000	152962963	40	413	413	5.9	0.27	–
Patel *et al.* [48]	2023	2005-2019	Canada	High-income North of America	305	1469880	14593301	–	305	305	20.75	2.09	–
Nielsen *et al.* [49]	2023	2004-2021	Denmark	Western Europe	7	63636	1000000	–	7	7	11	0.7	–
Xu *et al.* [50]	2024	2000-2023	China	East Asia	1358	58686441	–	–	1385		2.36		–
Lim and Kim [51]	2024	2014-2019	Republic of Korea	High-income Asia Pacific	888	59200000	296000000	–	888	888	1.5	0.3	–

### Global and regional incidence, prevalence, and mortality rate of PSC

**Fig 2:**
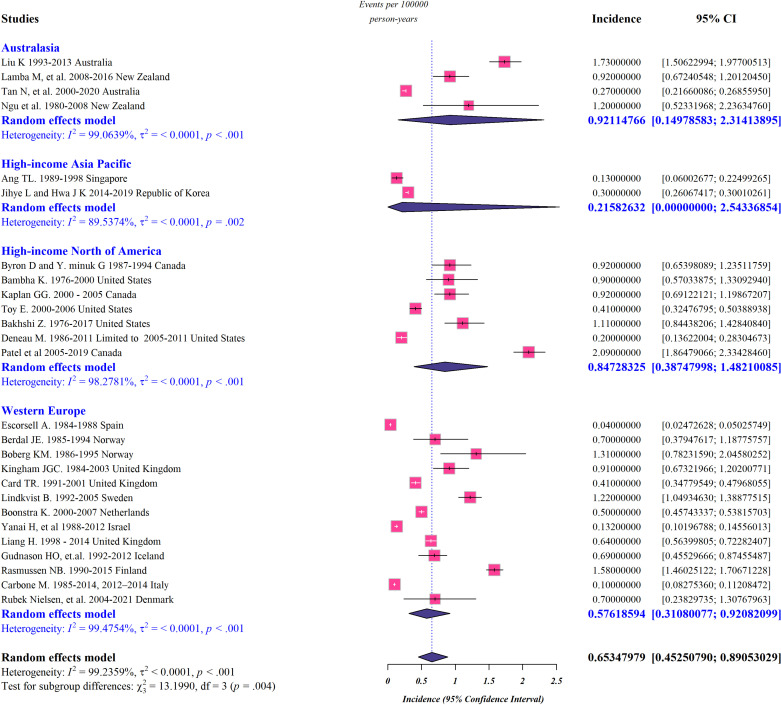
Forest plots representing the global and regional incidence rate of PSC.

**Fig 3:**
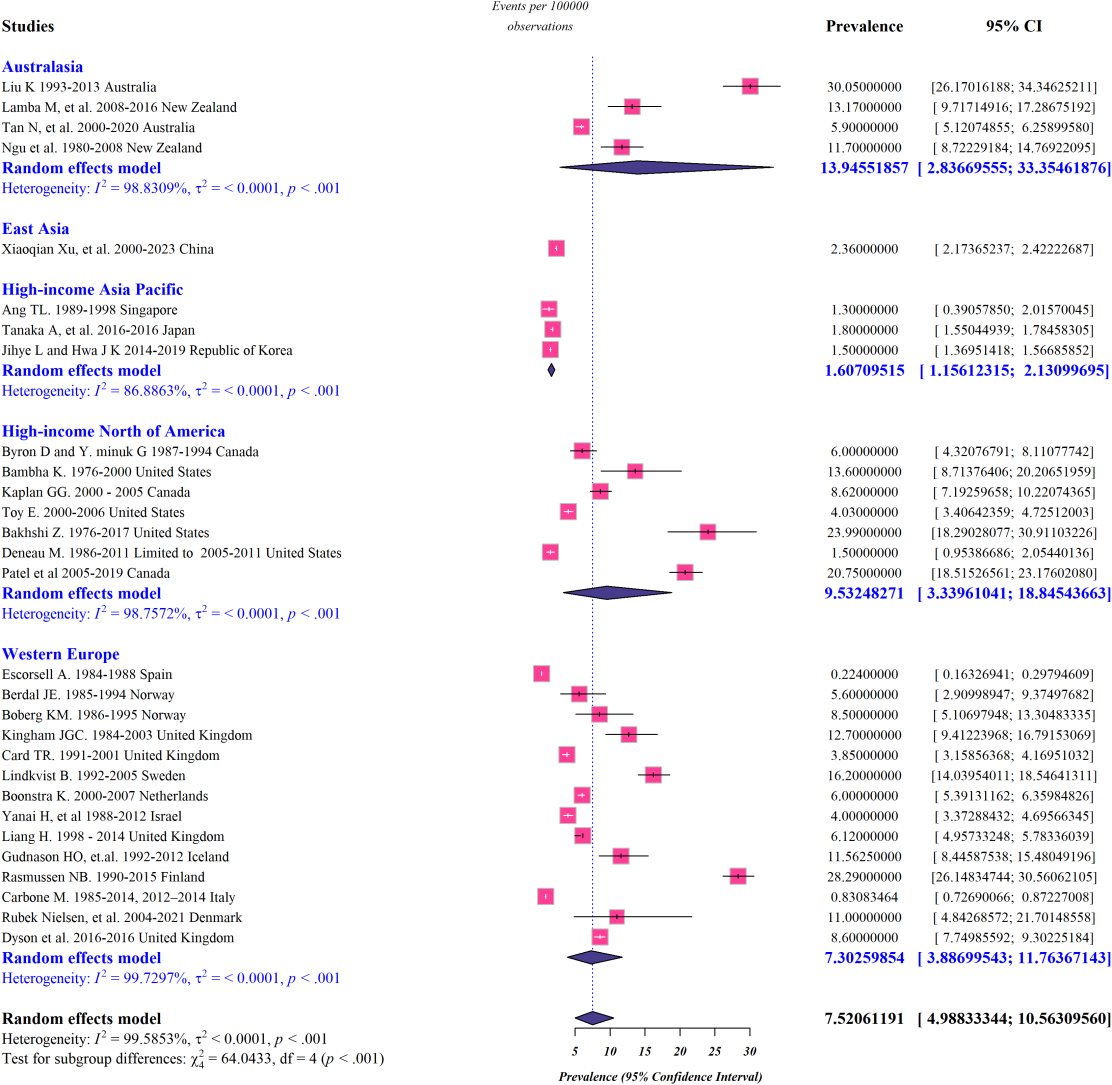
Forest plots representing the global and regional prevalence rate of PSC.

**Fig 4:**
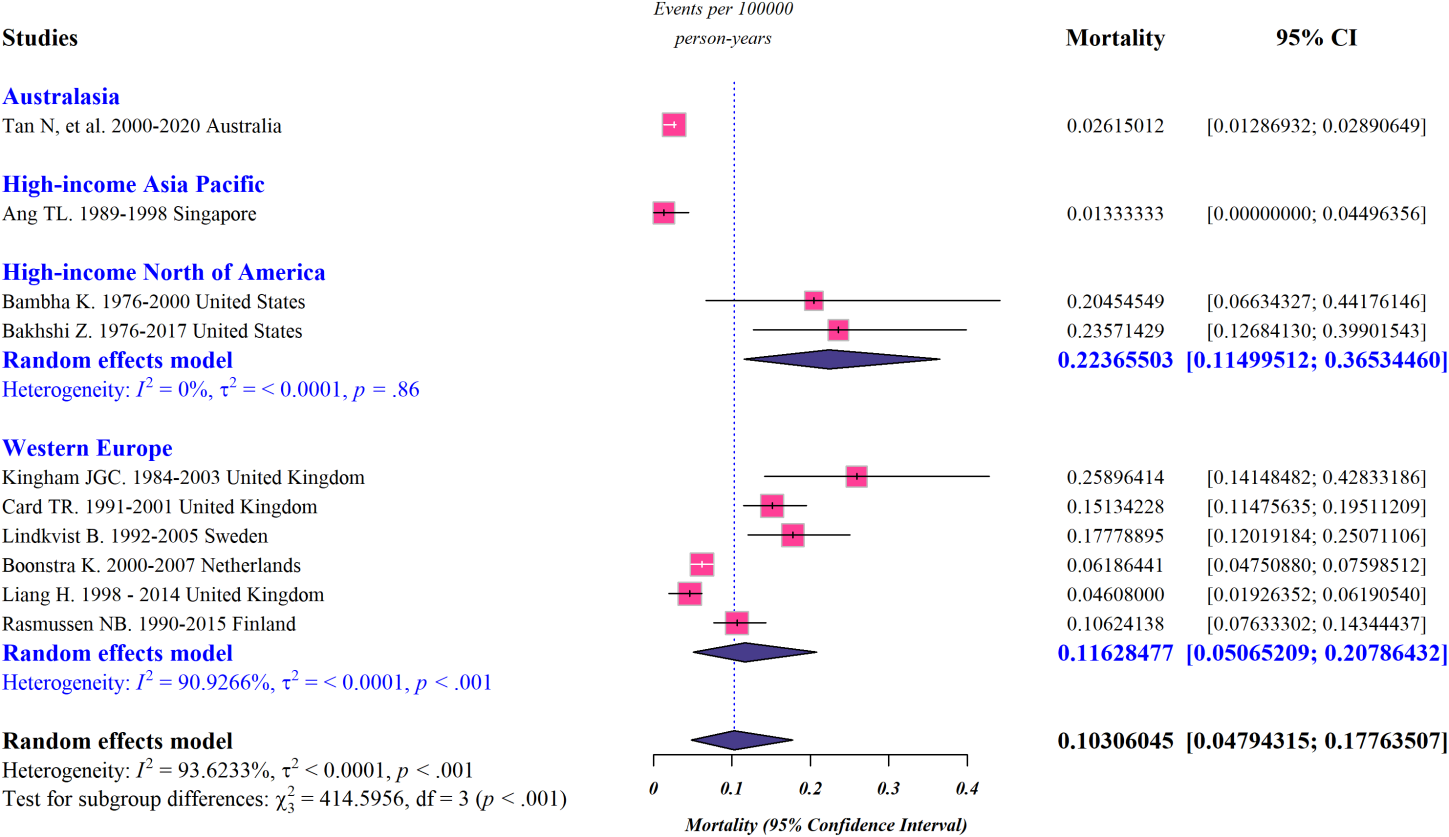
Forest plots representing the global and regional mortality rate of PSC.

Pooled subgroup analysis by region was performed for studies conducted in Australasia, high-income Asia Pacific, Northern America, and Western Europe, and the data representing incidence, prevalence, and mortality rate of PSC in these regions are presented in Figs 2-4. According to our meta-analysis, the worldwide pooled incidence rate of PSC was 0.65 per 100,000 persons (95% confidence interval [CI]: 0.45–0.89 per 100,000 persons; *I*^*2*^* *= 99.23%) (Fig 2). Additionally, the global-pooled prevalence and mortality rate of PSC were 7.52 per 100 000 persons (95% CI: 4.98–10.56 per 100,000 persons; *I*^*2*^* *= 99.58%) and 0.1 per 100,000 persons (95% CI: 0.04–0.17 per 100,000 persons; *I*^*2*^* *= 93.62%), respectively (Fig 3 and 4). The highest and lowest calculated prevalence of PCS was observed in Australasia (13.94 per 100,000 persons; 95% CI: 2.83–33.35 per 100,000 persons) and the high-income Asia Pacific (1.6 per 100,000 persons; 95% CI: 1.15–2.13 per 100,000 persons), respectively. Correspondingly, Australasia had the highest incidence rate of PCS, with 0.92 cases per 100,000 individuals (95% CI: 0.14–2.31 per 100,000 persons), while the lowest rate was seen in high-income Asia Pacific, with only 0.21 cases per 100,000 people (95% CI: 0–2.54 per 100,000 persons). Regional differences in the mortality rate of PSC appear to exist ranging from 0.01 per 100,000 persons (95% CI: 0–0.04 per 100,000 persons) in high-income Asia Pacific to 0.22 cases per 100,000 people (95% CI: 0.11–0.36 per 100,000 persons) in Northern America.

### Incidence, prevalence, and mortality rate of PSC across countries

Studies reporting the epidemiology of PSC within the population of Northern America have taken place between the years 1976 and 2019. Between the recorded period, the pooled incidence, prevalence, and mortality rate of PSC in the United States were 0.58 (95% CI: 0.09–1.47), 8.54 (95% CI: 0–31.18), and 0.22 (95% CI: 0.11–0.36) per 100,000 individuals, respectively. In Canada, the pooled incidence and prevalence of PSC were 1.26 (95% CI: 0.17–3.33) and 11.02 (95% CI: 0.31–36.6) per 100,000 persons. Population-based research on the epidemiology of PSC in Western Europe has covered a timeframe from 1984 to 2015. Accordingly, Spain and Italy were found to have the lowest incidence of PSC at a rate of 0.04 (95% CI: 0.02–0.05) and 0.1 (95% CI: 0.08–0.11) per 100,000 individuals, respectively, while Finland had the highest incidence at a rate of 1.58 per 100 000 people. As for prevalence, Spain and Italy had a rate of 0.22 (95% CI: 0.01–0.12) and 0.83 (95% CI: 0.74–0.89) per 100,000 persons, respectively, whereas Finland had a significantly higher rate of 28.28 (95% CI: 26.11–30.52) per 100,000 individuals.

A majority of epidemiological studies conducted on PSC in Europe have been carried out in the United Kingdom. The incidence and prevalence of PSC in the United Kingdom were 0.62 (95% CI: 0.15–1.38) and 7.3 (95% CI: 2.26–14.28) per 100,000 people, respectively, with a mortality rate of 0.13 (95% CI: 0–0.53) per 100,000 persons. Epidemiological studies are scarce on PSC in Asia, which makes it difficult to establish trends across the region with confidence. The Asian studies that met our inclusion criteria were conducted in the high-income Asia-Pacific region, including Singapore, Japan, the Republic of Korea, and China. We observed a significantly lower prevalence of PSC in Singapore compared to reported cases worldwide, with a rate of 1.3 (95% CI: 0.66–2.31) per 100,000 persons. Interestingly, our findings also suggest a relatively low incidence of PSC in Japan and the Republic of Korea, indicating a lower overall prevalence when compared to global statistics. Figs S3-S5 in S2 File illustrate the incidence, prevalence, and mortality rate of PSC across the countries examined, respectively.

### Projection

#### Future perspectives on the global prevalence of PSC.

**Fig 5:**
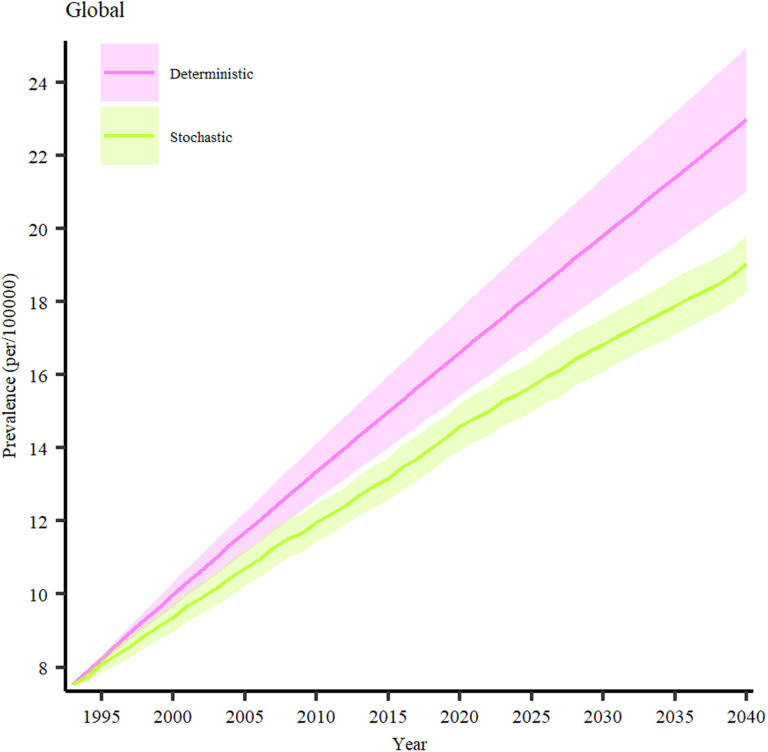
Prevalent PSC cases across the world from 1995–2040.

We forecasted the future prevalence of PSC in geographic areas with a minimum of three annual prevalence estimates derived from meta-analysis. This meticulous approach guaranteed the availability of ample data for robust modeling, thereby bolstering the precision of our prevalence estimates in these regions. Validation results are presented in Fig S6 in S2 File. According to our projections, the global prevalence of PSC is expected to increase significantly between 2024 and 2040 (Fig 5). Our analysis predicts that the prevalence will rise from 17.9 cases per 100,000 population (95% CI: 16.55–19.24) to 22.98 cases per 100,000 (95% CI: 21.0–24.95), representing an overall increase of 28.38%. This sharp upward trend highlights the growing burden of PSC on healthcare systems worldwide and underscores the importance of advancing research, early diagnosis, and treatment strategies to mitigate its impact in the coming decades.

#### Trends and future perspectives on the prevalence of PSC in Northern America and Western Europe.

**Fig 6:**
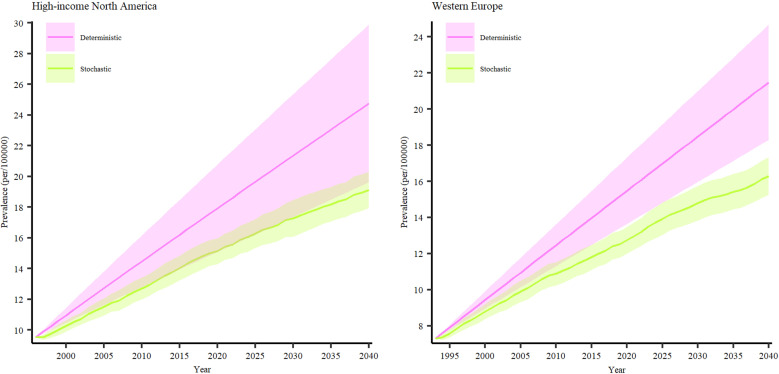
Prevalent PSC cases in High-income North America and Western Europe from 2019–2040.

Our findings indicate a substantial rise in the prevalence of PSC in North America and Western Europe between 2024 and 2040. Specifically, North America is projected to experience a 5.45% increase in PSC cases, while Western Europe is expected to see a 28.79% rise by 2040 (Fig 6). In North America, the prevalence is estimated to grow from 19.3 cases per 100,000 (95% CI: 16–22.6) in 2024 to 24.76 cases per 100,000 (95% CI: 19.63–29.88) by 2040. Similarly, in Western Europe, the prevalence is projected to increase from 16.67 cases per 100,000 (95% CI: 14.55–18.77) in 2024 to 21.48 cases per 100,000 (95% CI: 18.3–24.65) by 2040. In our study, we further focused on three countries situated in North America and Western Europe, namely the United States, the United Kingdom, and Canada. In our investigation, we honed in on three countries located in North America and Western Europe: the United States, the United Kingdom, and Canada. These specific nations were chosen based on the existence of at least three published annual estimates regarding PSC prevalence. The percentage changes in PSC prevalence from 2024 to 2040 indicate a forecasted increase of about 10.4% in the United States, 21.12% in the United Kingdom, and 15.75% in Canada. Consequently, our projections suggest that the prevalence rates in 2040 will be 10.82 cases per 100,000 (95% CI: 9.43–12.21) for the United States (Figs S7 in S2 File), 12.05 cases per 100,000 (95% CI: 9.14–14.96) for the United Kingdom (Figs S8 in S2 File), and 16.06 cases per 100,000 (95% CI: 13.52–18.6) for Canada (Fig S9 in S2 File).

#### Trends and future perspectives on the prevalence of PSC in Asia-Pacific and Australia.

**Fig 7:**
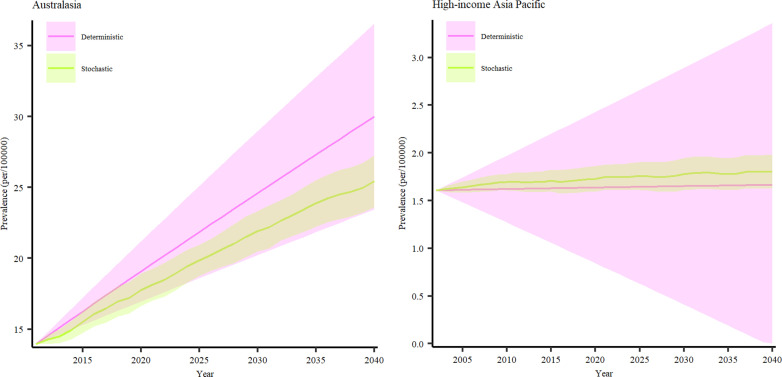
Prevalent PSC cases in Australasia and High-income Asia-Pacific from 2019–2040.

Our findings also revealed a rising trend in the prevalence of PSC in the Asia-Pacific and Australasia regions between 2024 and 2040 (Fig 7). The projected prevalence in the high-income Asia-Pacific region shows a modest increase of approximately 1.35%, whereas Australia is expected to experience a dramatic rise of 40.84%. Specifically, in Australia, PSC prevalence is projected to grow from 21.29 cases per 100,000 (95% CI: 18.26–2.61) in 2024–30 cases per 100,000 (95% CI: 23.45–36.54) in 2040. In Asia-Pacific, the prevalence is expected to increase from 1.64 cases per 100,000 (95% CI: 0.67–2.61) in 2024 to 1.66 cases per 100,000 (95% CI: 0–3.35) by 2040 (Table 2).

**Table 2:** The estimated prevalence of PSC projected in our study across the world from 2025-2040.

Region (country)	Prevalence per 100,000 population				Change percentage (%)
	2025	2030	2035	2040	2024–2040
Global	18.21 (95% CI: 16.83–19.6)	19.81 (95% CI: 18.22–21.39)	21.39 (95% CI: 19.61–23.17)	22.98 (95% CI: 21–24.95)	28.38
High-income North of America	19.64 (95% CI: 16.22–23.06)	21.35 (95% CI: 17.36–25.34)	23.06 (95% CI: 18.5–27.62)	24.76 (95% CI: 19.63–29.88)	5.45
United States of America	9.86 (95% CI: 9.02–10.7)	10.18 (95% CI: 9.15–11.2)	10.5 (95% CI: 9.29–11.7)	10.82 (95% CI: 9.43–12.21)	10.38
Canada	14.01 (95% CI: 12.47–15.54)	14.69 (95% CI: 12.82–16.56)	15.37 (95% CI: 13.16–17.58)	16.06 (95% CI: 13.52–08.60)	15.75
Western Europe	16.97 (95% CI: 14.81–19.14)	18.48 (95% CI: 15.98–20.98)	19.98 (95% CI: 17.14–22.82)	21.48 (95% CI: 18.3–24.65)	28.79
United Kingdom	10.08 (95% CI: 8.36–11.8)	10.74 (95% CI: 8.62–12.85)	11.39 (95% CI: 8.88–13.91)	12.05 (95% CI: 9.14–14.96)	21.12
Australasia	21.85 (95% CI: 18.59–25.11)	24.59 (95% CI: 20.22–28.96)	27.31 (95% CI: 21.84–32.77)	30.00 (95% CI: 23.45–36.54)	40.84
High-income Asia Pacific	1.643 (95% CI: 0.62–2.65)	1.650 (95% CI: 0.41–2.89)	1.657 (95% CI: 0.19–3.12)	1.664 (95% CI: 0.1–3.35)	1.35

## Discussion

Herein, we conducted a comprehensive analysis of the global incidence, prevalence, and mortality rates of PSC, utilizing an IDM approach to project future trends based on current data. Our findings demonstrate a significant rise in the number of individuals diagnosed with PSC in recent years, indicating that the disease may impose a substantial public health burden going forward. Although PSC is categorized as a rare disease, its prevalence is marked by regional variability, with notably higher incidence rates observed in Western countries—such as North America and Northern Europe—compared to those in Asia. This disparity underscores the complex interplay of both known and unidentified factors contributing to the heterogeneity in PSC epidemiology. Such variations reflect distinct regional differences in disease prevalence and distribution patterns.

Our meta-analysis revealed considerable variability in PSC prevalence and incidence rates across various global regions. Specifically, we found prevalence rates ranging from 1.6 cases per 100,000 persons in the high-income Asia-Pacific region to 13.94 cases per 100,000 persons in Australasia. Similarly, incidence rates varied significantly, from 0.21 cases per 100,000 inhabitants per year in the high-income Asia-Pacific region to 0.92 cases per 100,000 inhabitants per year in Australasia. Notably, our analysis indicated that Western European countries exhibit the highest prevalence and incidence of PSC, particularly in Northwestern countries and Scandinavia, in contrast to the Mediterranean basin. These observations align closely with existing literature, corroborating the higher prevalence of PSC in Western regions [9,20,21]. Collectively, our findings highlight the urgent need for enhanced surveillance and research into PSC, particularly in regions where its impact may be escalating. Continued investigation into the underlying factors contributing to these geographic disparities will be essential for developing targeted prevention and treatment strategies.

Several population-based and environmental determinants are believed to significantly influence the global epidemiology of PSC. These determinants include genetic predisposition, lifestyle choices, dietary patterns, and sunlight exposure. Notably, urbanization and the adoption of Westernized lifestyles have been linked to an increasing prevalence of autoimmune disorders [22,23], supporting the idea that Australasia may experience a burden of PSC comparable to that observed in well-developed Northern Hemisphere nations. In contrast, there is a concerning scarcity of robust population-based epidemiological studies on PSC in regions such as Asia, South America, and Africa, especially within developing countries. This lack of data raises important questions regarding potential referral bias and underreporting. While limited studies have been conducted in Japan and Singapore, their methodologies for case identification have often been inadequate, which hampers our ability to draw definitive conclusions about PSC prevalence in these areas. It is also important to consider that the higher prevalence of reported PSC cases in Western regions may be linked to better healthcare accessibility and advanced diagnostic technologies. This enhanced capacity for detection has likely allowed for the identification of subclinical cases of sclerosing cholangitis, potentially leading to an overestimation of incidence rates in high-income Western countries compared to other regions. Overall, a more comprehensive understanding of PSC epidemiology requires additional, rigorous population-based studies in underrepresented regions to address these disparities and improve our grasp of the disease’s true global burden.

Our modeling depictes a notable global upsurge in the prevalence of PSC, projecting a prevalence of 22.98 cases per 100,000 by the year 2040, indicating a substantial increase of 28.3%. Our estimations underscore a substantial escalation in the population affected by PSC in the future, pointing towards a looming burden of this condition in the future. Furthermore, the findings unveil a marked elevation in PSC prevalence in both Australia and Western Europe by 2040. However, it is important to consider that the available data on PSC prevalence and projections are based on limited studies and may not fully capture the true epidemiology of the disease. True population-based epidemiological data on PSC are limited in Asia, South America, and Africa, thus, the prevalence of PSC in developing countries is likely underestimated. Moreover, limited access to advanced healthcare and challenges in confirming the diagnosis without specific procedures like endoscopic retrograde cholangiopancreatography (ERCP) can impact the burden of PSC reported from developing countries. In addition, our estimation can be influenced by various factors such as changes in diagnostic criteria, increased awareness, improved detection methods, and changes in population demographics. Therefore, it is imperative to highlight that these factors are speculative, and further research is necessary to fully understand their potential impact on the burden of PSC in the future.

While interpreting our study, it is important to acknowledge its limitations. Firstly, our findings may have been impacted by the limited number of eligible studies in certain areas and the substantial heterogeneity among the included studies, potentially affecting our estimation of uncertainty. Secondly, the lack of accessibility to relevant data regarding sex and age group variables prevented us from conducting projections based on these factors. Thirdly, our study focused exclusively on English language reports concerning PSC, potentially resulting in the omission of certain epidemiological data. Nevertheless, as highlighted in a study by Nussbaumer-Streitet et al [24]., the choice of language in the selected studies was noted to have no discernible impact on the precision and reliability of the findings. Lastly, while our projections extended to 2040, we acknowledge that longer-term forecasts may carry increased uncertainty and a heightened risk of overestimation, primarily due to the constraints of deterministic modeling approaches.

In conclusion, our findings reveal a substantial increase in the number of individuals diagnosed with PSC in recent years, which suggests a potential escalation of the disease’s public health burden in the future. However, it is essential to interpret these results with caution, given the limitations related to data availability, potential confounding factors, and the pressing need for further research to accurately assess PSC’s global impact. Studying PSC on a global scale is inherently challenging due to its rarity and the lack of standardized diagnostic criteria. Furthermore, disparities in healthcare access, referral patterns, and clinical practices may significantly affect the accuracy and comparability of epidemiological data across different regions. The dearth of population-based studies in certain areas, particularly in developing countries, further exacerbates the challenge of fully understanding the true global burden of PSC. To address these gaps, future research should prioritize conducting large-scale, population-based studies in diverse geographic regions, with particular focus on underrepresented areas such as Asia, South America, and Africa. These studies should implement standardized diagnostic criteria, employ rigorous case-finding methodologies, and utilize comprehensive data collection approaches to enhance the accuracy and comparability of epidemiological estimates. By doing so, we can advance our understanding of PSC and its impact on global health, ultimately facilitating more effective prevention and treatment strategies.

## Supporting information

S1 File Literature search results and extracted data. (XLSX)

S2 File Supplementary figures and plots of this study. (PDF)

S3 File R Scripts and Code Utilized in This Study. (PDF)
