# Supplementary material for: The epidemiological trends and projected future of primary sclerosing cholangitis by 2040: An updated meta-analysis and modeling study
Source: PLoS One. 2025 May 5;20(5):e0322479. doi: 10.1371/journal.pone.0322479 (PMC12052114; doi:10.1371/journal.pone.0322479)

The Epidemiological Trends and Projected Future of Primary Sclerosing Cholangitis by 2040: An Updated Meta-Analysis and Modeling Study Worldwide

(Supporting Information)

Figure S1. Egger's plot to assess the publication bias.

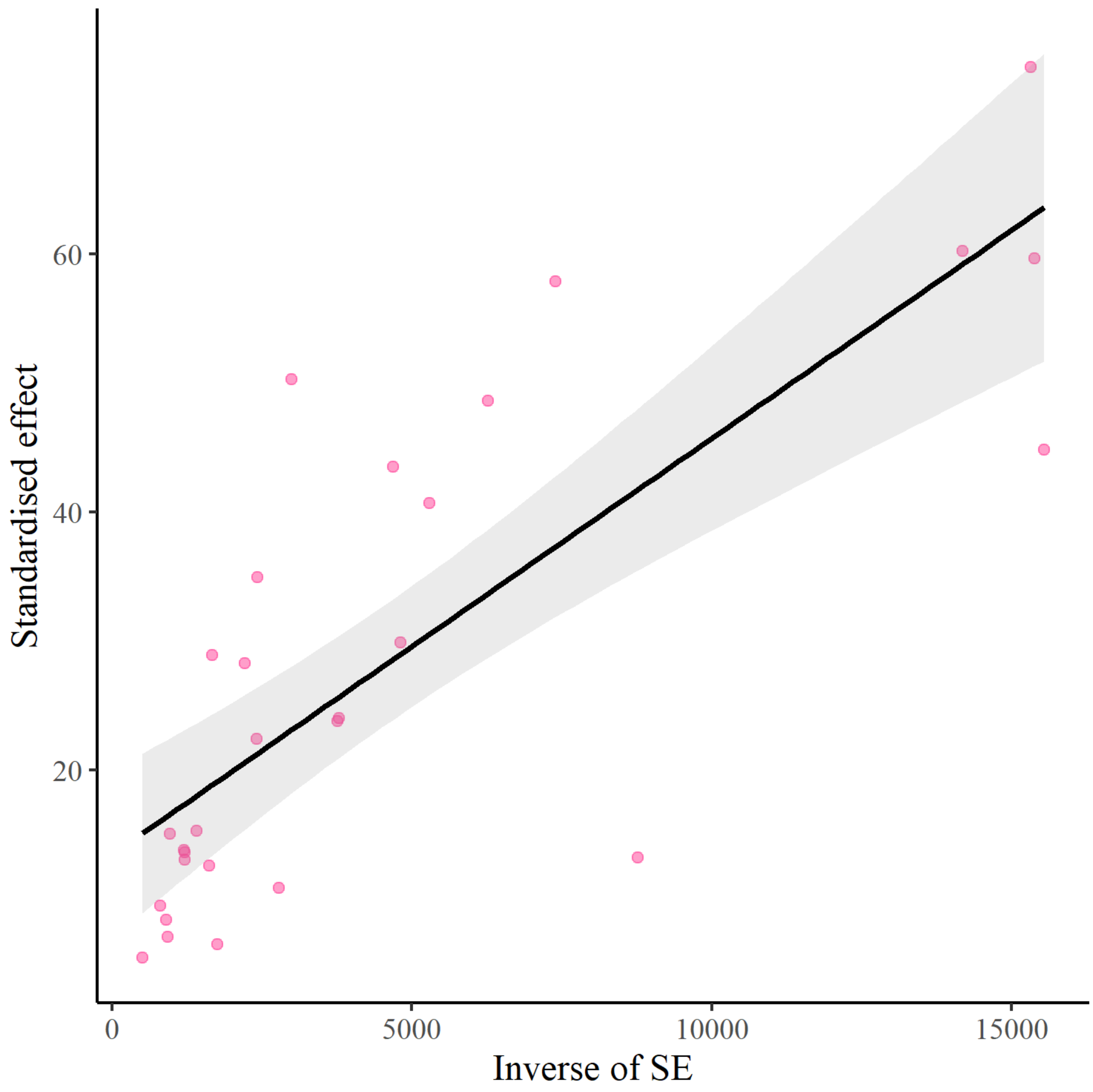

Figure S2. Doi plot for the pooled prevalence of PSC. A Luis Furuya- Kanamori (LFK) index of 6.6 indicates major asymmetry.

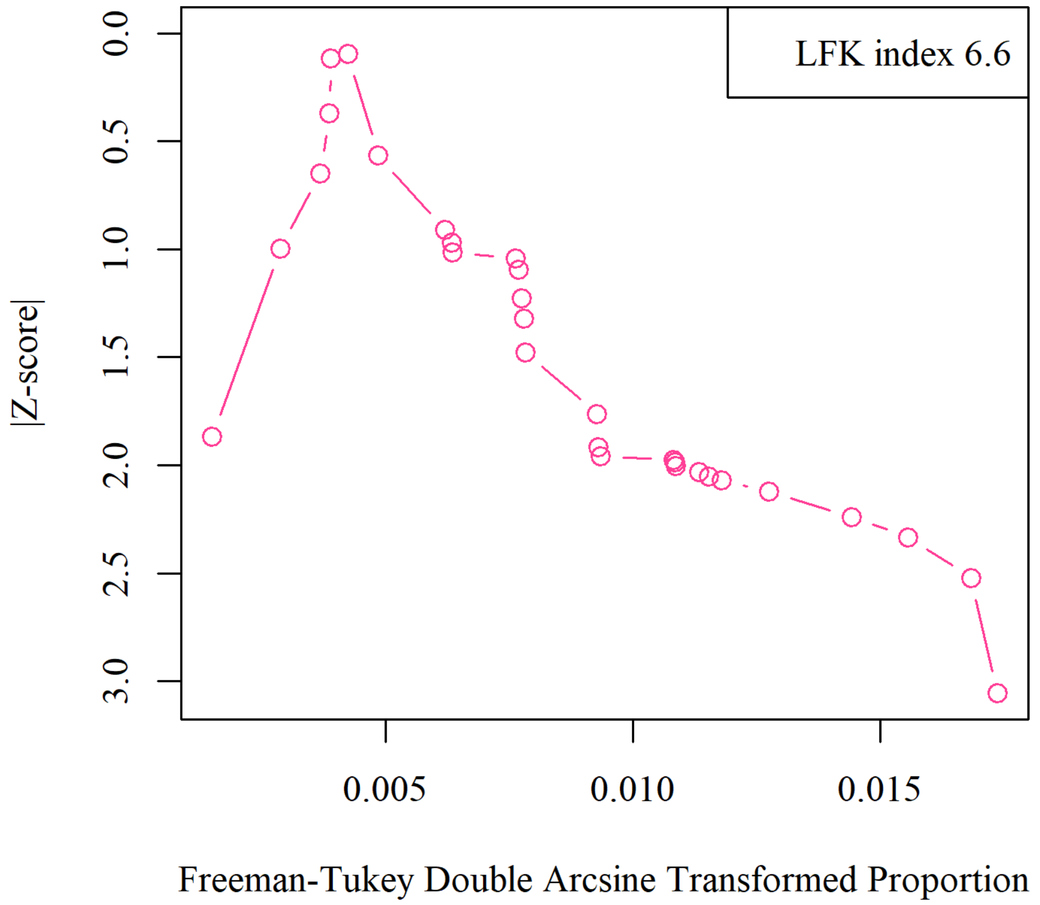

Figure S3. Forest plots representing the incidence of PSC across the countries.

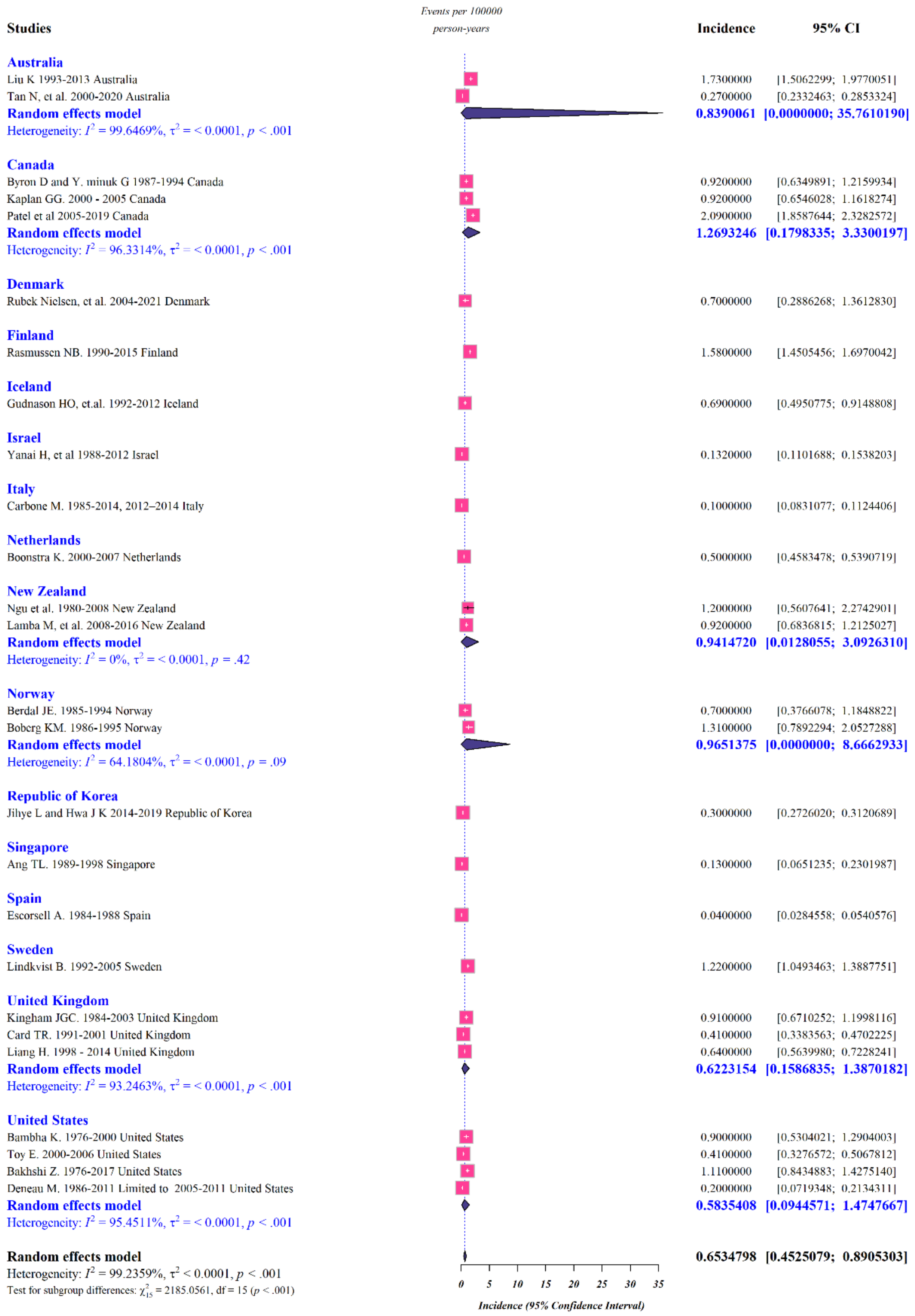

Figure S4. Forest plots representing the prevalence of PSC across the countries.

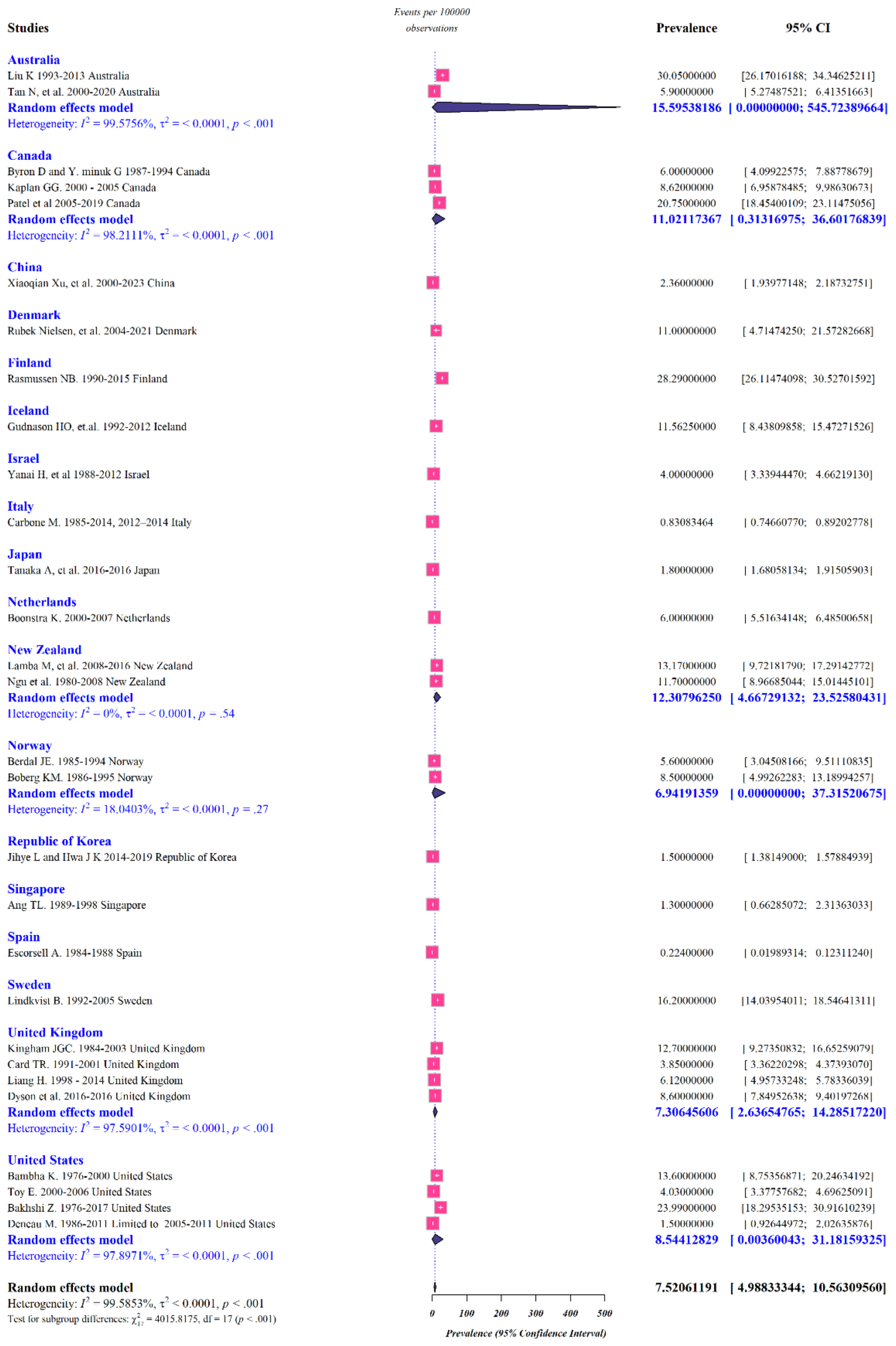

Figure S5. Forest plots representing the prevalence of PSC across the countries.

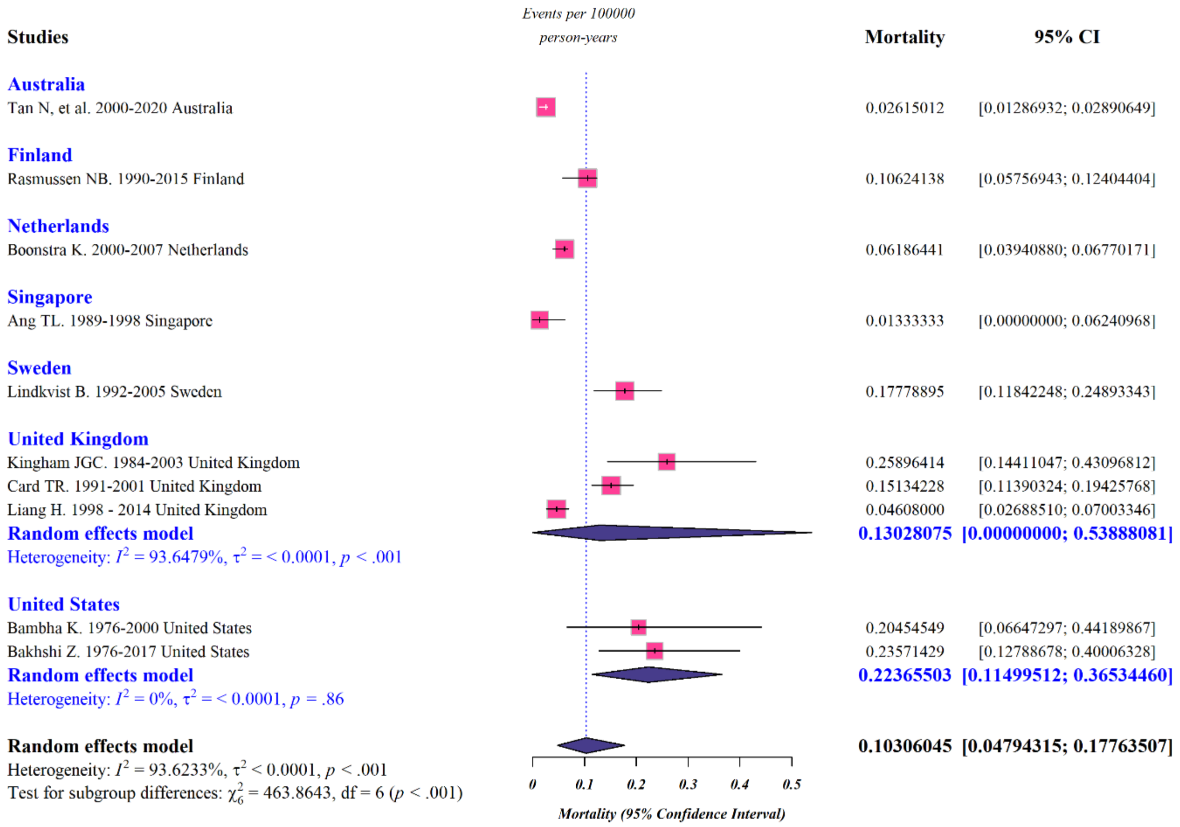

Figure S6. Validation results of the IDM model to the calculated annual prevalence data for each geographical unit and refined the model parameter values using the Limited-memory BFGS.

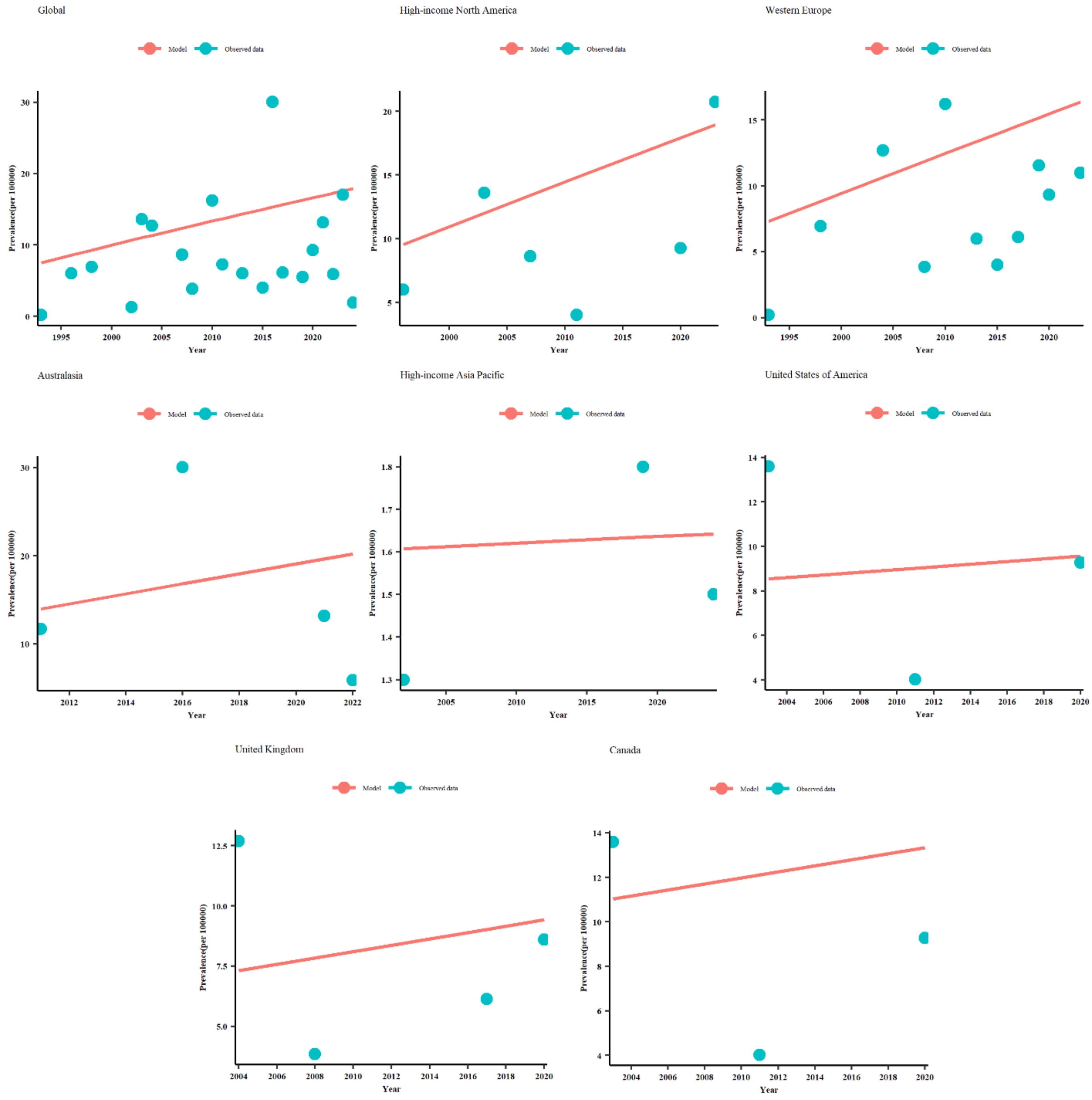

Figure S7. Prevalent PSC cases in the United States from 2024–2040.

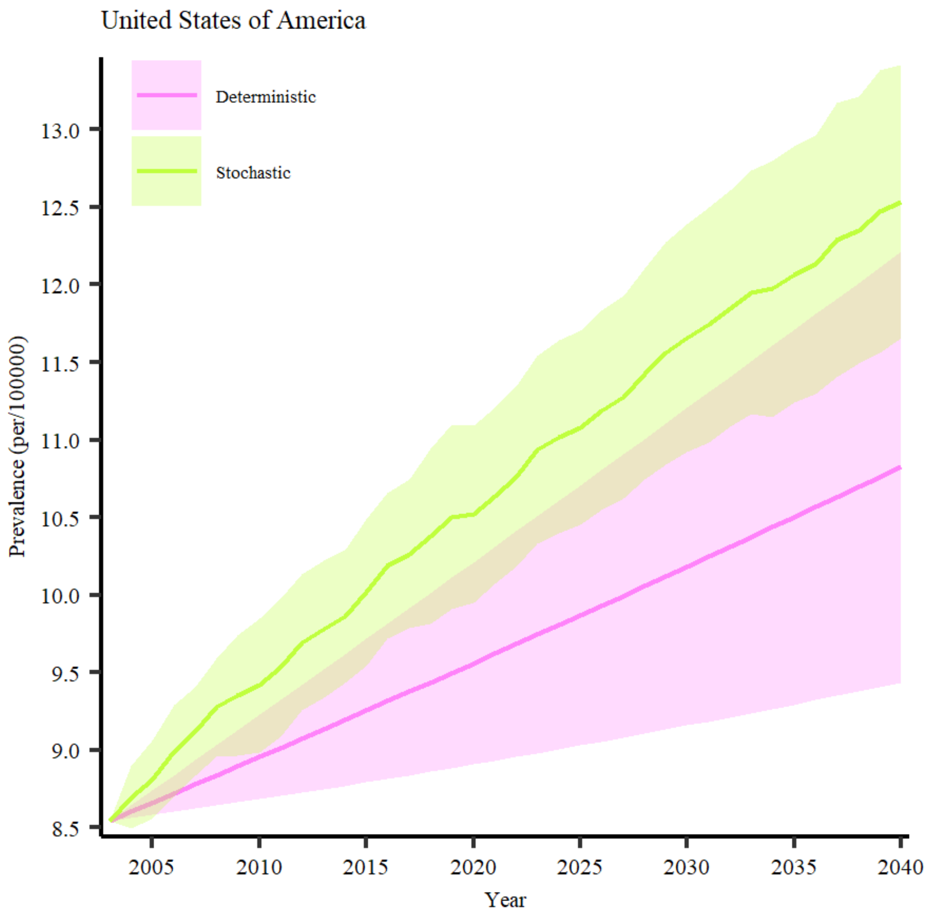

Figure S8. Prevalent PSC cases in the United Kingdom from 2024–2040.

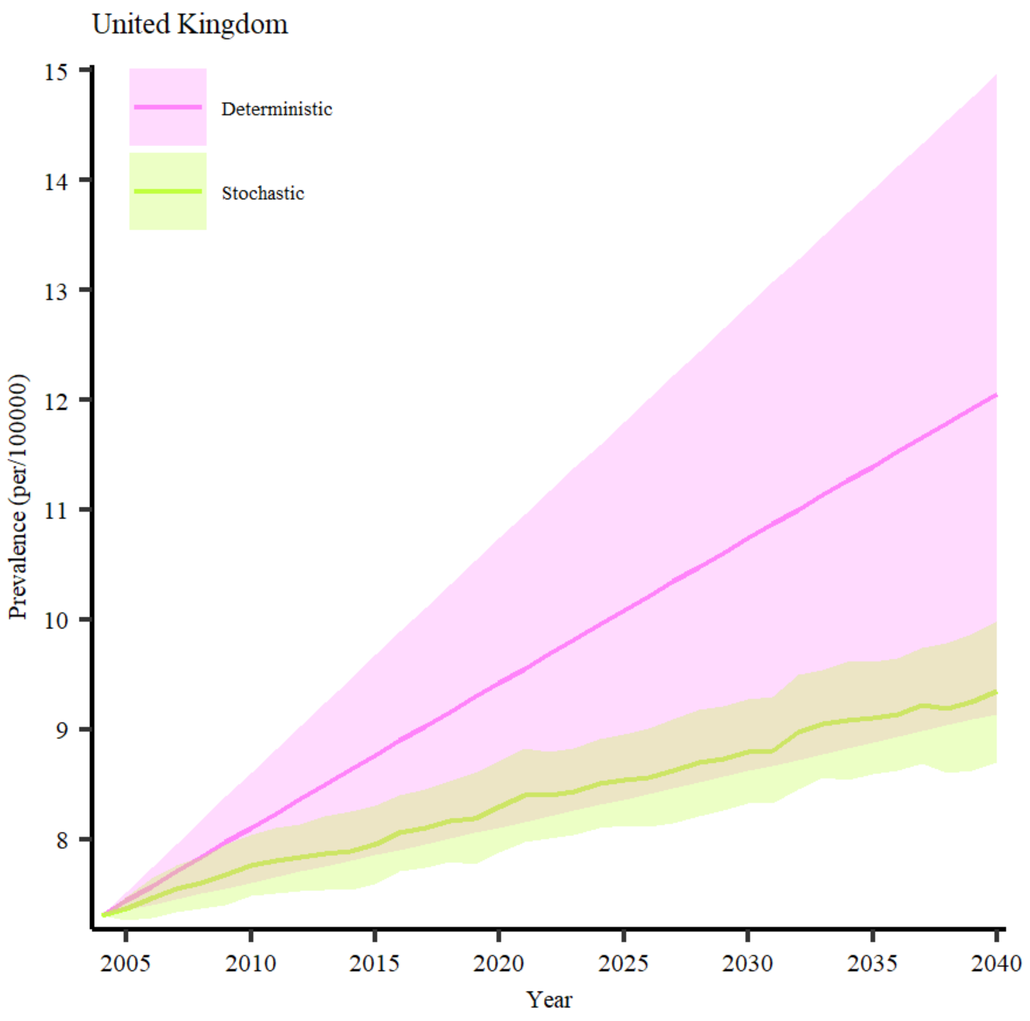

Figure S9. Prevalent PSC cases in Canada from 2024–2040.

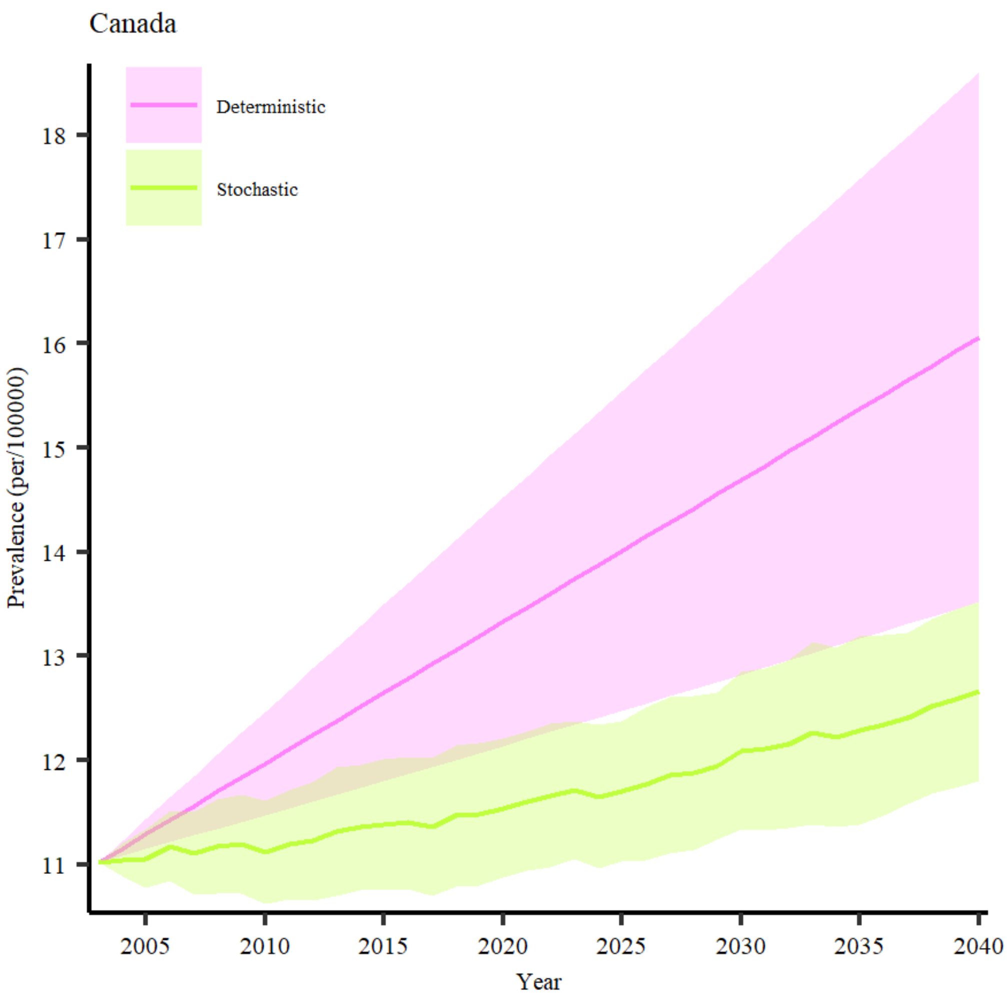

Supplement: S2 File — (PDF) [file pone.0322479.s002.pdf]
