# Supplementary material for: The epidemiological trends and projected future of primary sclerosing cholangitis by 2040: An updated meta-analysis and modeling study
Source: PLoS One. 2025 May 5;20(5):e0322479. doi: 10.1371/journal.pone.0322479 (PMC12052114; doi:10.1371/journal.pone.0322479)
Supplement: S3 File — (PDF) [file pone.0322479.s003.pdf]

```
##############################
#######Sample code for World data by Meysam Olfatifar
####### For other regions the same process should be repeated
##############################
rm(list = ls())
library(deSolve)
library(FME)
library(ggplot2)
library(dplyr)
library(xlsx)
library(readxl)
# Birth number, population and total mortality rate for each region/ country from GBD database
birth<-read.csv("")
pop<-read.csv("")# from GBD data
pop1<-read.csv(".../IHME_POP_2017_2100_POP_REFERENCE_Y2020M05D01.csv")
Death<-read.csv("...../IHME-GBD_2019_DATA-8e5a79c2-1.CSV")



###Specified data based on meta analysis

#Incidence rate estimated by meta analysis
in.mat<-read_excel(".../Regional_results.xlsx",1)
in.mat<-as.data.frame(in.mat)

# Total prevalence rate estimated by meta analysis

p.mat<-read_excel(".../Regional_results.xlsx",2)
p.mat<-as.data.frame(p.mat)
```

```
#Specific mortality rate estimated by meta analysis
de.mat<-read_excel("...//Regional_results.xlsx",3)
de.mat<-as.data.frame(de.mat)

#Annual prevalence data for validation obtained through meta-analysis

validation<-read_excel("G://Works in progress//Projection//PSC//Prevalence_Data.xlsx",5)

# Preparing the birth and population data
birth$scenario_name<-birth$scenario_name%>%
  na_if("")
birth$scenario_name<-ifelse(is.na(birth$scenario_name),paste("Post"),birth$scenario_name)
birth<-birth[with (birth, scenario_name== "Reference" | scenario_name== "Post")&
            birth$year_id==2019,
          c("year_id","location_name","val" ,"upper", "lower")]

popfe<-pop[pop$sex=="Female"& pop$age_group_name=="All Ages"&
          with(pop,year_id >=1990 & year_id <=2019),
         c("year_id","location_name","val")]
popma<-pop[pop$sex=="Male"& pop$age_group_name=="All Ages"&
          with(pop,year_id >=1990 & year_id <=2019),
         c("year_id","location_name","val")]
pop<-cbind(popfe[,3] + popma[,3])
pop<-cbind(pop,popfe[,1:2])

pop1fe<-pop1[pop1$sex=="Female" & pop1$age_group_name=="All Ages"&
            with(pop1,year_id >=2018 ),
          c("year_id","location_name","val")]
```

```
pop1ma<-pop1[pop1$sex=="Male" & pop1$age_group_name=="All Ages"&
          with(pop1,year_id >=2018),
        c("year_id","location_name","val")]

pop1<-cbind(pop1fe[,3] + pop1ma[,3])
pop1<-cbind(pop1,pop1fe[,1:2])

names(pop)[1]<-c("val")
names(pop1)[1]<-c("val")
pop<-rbind(pop,pop1)

######################

pars<- list( BirthNumber=birth[birth$location_name=="Global","val"],
        DeathNonPSC= Death[Death$location_name=="Global","val"],
        DeathPSC=de.mat[de.mat$Location=="Global",  "events"],
        PSCincidence=in.mat[in.mat$Location=="Global","events"])
parRanges <- data.frame(min = c( birth[birth$location_name=="Global","lower"],
                Death[Death$location_name=="Global","lower"],
                de.mat[de.mat$Location=="Global","lower_ci"]
                , in.mat[in.mat$Location=="Global","lower_ci"]),
             max = c( birth[birth$location_name=="Global","upper"],
                Death[Death$location_name=="Global","upper"],
                de.mat[de.mat$Location=="Global","upper_ci"]
                , in.mat[in.mat$Location=="Global","upper_ci"]))
rownames(parRanges) <- c("BirthNumber","DeathNonPSC","DeathPSC",
             "PSCincidence")
parRanges
```

```
popu<-pop[pop$year_id=="2019"& pop$location_name=="Global","val"]

prev<-(p.mat[p.mat$Location=="Global", "events"]*popu)/100000


# Model function

model <- function(pars, time) {

 derivs <- function(t, state, pars) {

  with(as.list(c(state, pars)), {

   # Adjusted calculations for deaths and incidence for better realism

   death <- (DeathNonPSC * Health) / 100000  # Proportional mortality

   incidence <- (PSCincidence * (Health - PSC)) / 100000  # Incidence based on susceptible
population


   # Calculating total health exits

   hExit <- death + incidence


   # Change in health state and prevalence of pulmonary cases

   dHealth <- BirthNumber - hExit  # Population changes from births and exits

   dPSC <- incidence - (DeathPSC * PSC) / 100000  # Change in pulmonary cases


   # Return the rates of change and the prevalence

   return(list(c(dHealth, dPSC),

         Prevalence = (PSC * 100000) / (Health)))

  })

 }


 # Initial state

 state <- c(Health = popu - prev, PSC = prev)
```

```r
  # ODE solves the model by integrating over time
  return(as.data.frame(ode(y = state, times = time, func = derivs, parms = pars)))
}




objective_function <- function(pars) {
  model_output <- model(pars, time)

  # Interpolate validation data to match the model output times
  interpolated_events <- approx(validation$Year, validation$events, xout = model_output$time)$y

  # Calculate difference between observed (interpolated) and modeled prevalence
  diff <- model_output$Prevalence - interpolated_events

  # Return the sum of squared differences
  return(sum(diff^2, na.rm = TRUE))  # use na.rm=TRUE to avoid issues with NAs if lengths differ
}

# Initial parameter guesses
initial_pars<- c( BirthNumber=birth[birth$location_name=="Global","val"],
          DeathNonPSC= Death[Death$location_name=="Global","val"],
          DeathPSC=de.mat[de.mat$Location=="Global",  "events"],
          PSCincidence=in.mat[in.mat$Location=="Global","events"])


time <- seq(min(validation$Year), max(validation$Year), by = 1)  # Create a proper time sequence
```

```
# Optimization to fit parameters
fit <- optim(
  par = pars,
  fn = objective_function,
  method = "L-BFGS-B",
  lower = parRanges$min,
  upper = parRanges$max
)

# Results
fit_params <- fit$par
fit_params
# Generate fitted model output using the optimized parameters
fitted_model <- model(fit_params, time)  # Use the fitted parameters from optimization

library(ggplot2)  # For plotting

# (Assume the previous code to define the model, observed data, and fitting is already run here)




# Interpolate the observed prevalence to match the fitted model's time points
observed_interpolated <- approx(validation$Year, validation$events, xout = fitted_model$time)$y

# Assuming validation has a Year column representing the time
plot_data <- data.frame(
  time = validation$Year,
  observed_prevalence = validation$events
)
```

```
# Add fitted model prevalence only for those time points present in validation

# Merge the fitted model data with the validation data

plot_data <- merge(plot_data,

          data.frame(time = fitted_model$time,

               fitted_prevalence = fitted_model$Prevalence),

          by = "time",

          all.x = TRUE)


tiff("Validation_Global.tiff", width = 3,

  height =3, units = 'in', res = 300,compression = c( "lzw") )

# Create the plot

ggplot(plot_data, aes(x =time))+

 geom_line(aes(y=fitted_prevalence, color = "Model"),size = 1) +

 geom_point(aes(y = observed_prevalence, color = "Observed data"), size = 3)+# Add lines for fitted
and observed

 # Add points for observed values

 labs(title = "Global",

   x = "Year",

   y = "Prevalence(per 100000)",

   color = "Legend") +

 theme_classic()+# Adjusting y-axis limit

 theme(text=element_text(size=5, family="serif")) +

 theme(legend.position = "top", axis.text.x = element_text(face = "bold",color = "black", size =5),

    axis.text.y = element_text(face = "bold",color = "black",size = 5

    ))+ theme(legend.title = element_blank())+

 theme(axis.text=element_text(size=5,face="bold"),

    axis.title=element_text(size=5,face="bold"))+

 scale_x_continuous(breaks = scales::pretty_breaks(n = 8), expand = c(0.01,0))
```

```
dev.off()

##############################Projection

# Assuming the forecast is generated as before
previous_length <- length(time)
last_year <- 2040
start_year <- max(plot_data$time)
forecast_period <-seq(start_year + 1, last_year)  # Start from the next year after the last data point

# Calculate the total project time for the forecast
total_project_time <- length(forecast_period)+length(time)

# Generate the forecast using the model function
forecast_data <- model(fit_params, time = 1:total_project_time)
time1<-min(plot_data$time):last_year
# Create a data frame to hold the new forecast data
forecast_df <- data.frame(
  time = time1,
  forecast = forecast_data  # Assuming forecast_data is a vector of the same length as
forecast_period
)

## Bootstrap technique to estimate 95% confidence interval
bootstrap_params <- function(data, n_boot = 100) {
  results <- matrix(NA, nrow = n_boot, ncol = length(pars))
  for (i in 1:n_boot) {
    sample_indices <- sample(nrow(data), replace = TRUE)  # Sample with replacement
    sample_data <- data[sample_indices, ]  # Full sample data for model fitting
```

```
    fit <- optim(
      par = pars,
      fn = objective_function,
      method = "L-BFGS-B",
      lower = parRanges$min,
      upper = parRanges$max
    )
    results[i, ] <- fit$par
  }
  return(results)
}

# Execute bootstrap with the full validation dataframe
boot_results <- bootstrap_params(validation)
# Calculate confidence intervals
cis <- apply(boot_results, 2, quantile, probs = c(0.025, 0.975))

colnames(cis)<-c("BirthNumber","DeathNonPSC","DeathPSC",
            "PSCincidence")

row.names(cis)<-c("min","max")

cis<-as.data.frame(t(cis))
# Ensure that cis is a list of parameters
cis_list <- split(cis, rownames(cis))
model_with_time <- function(pars) {
  model(pars, time1)
}
```

```
global1 <- summary(sensRange(func = model_with_time, parms = fit_params,
                     dist = "latin", sensvar = "Prevalence" ,
                     parRange = cis, num = 100))
z<-sensRange(func = model_with_time, parms = fit_params,
          dist = "latin", sensvar = "Prevalence",
          parRange = parRanges, num = 100)



z<-z[,-c(1:4)]
se<- function(x) sd(x) / sqrt(length(x))
s<-apply(z,2,se)*1.96
r<-data.frame(global1,s)
```
